# Fast and richly structured activity in cortical networks with local inhibition

**DOI:** 10.1186/1471-2202-12-S1-P121

**Published:** 2011-07-18

**Authors:** Guillaume Hennequin, Tim Vogels, Wulfram Gerstner

**Affiliations:** 1 School of Computer and Communication Sciences, Brain-Mind Institute, Ecole Polytechnique Fédérale de Lausanne, 1015 Lausanne EPFL, Switzerland

## 

Spiking models of small areas of the neocortex have so far mainly been studied under the assumption that neurons are sparsely and randomly connected. The absence of structure allows for the use of powerful mean-field approximation techniques, whereby statistical properties of spike trains such as the average firing rate and the interspike interval coefficient of variation are described in terms of their distributions across neurons [1,2]. Such approaches cannot offer a more detailed description as to what patterns of activity the network spontaneously produces. The macroscopic state is unstructured with predictable mean, while during the asynchronous irregular firing the microscopic dynamics is chaotic. It therefore becomes hard to think of the representation of information in cortex if its activity is neither structured nor reproducible.

Connectivity in cortex is not purely random: published data on synaptic connectivity suggests a model of cortical connectivity in which excitatory neurons make long-range patchy projections while inhibitory neurons only target their close neighbours [3]. How does this structure affect the dynamics of the network? Can we hope to describe it in terms of activity patterns, therefore going beyond the mean-field predictions mentioned above? Recent theoretical considerations [4] have suggested a positive answer, by showing that balanced networks should be expected to transiently amplify certain activity patterns without slowing the dynamics down (“balanced amplification”). Underlying this theory is the nature of the connectivity matrix, “nonnormal” in the mathematical sense [5]. The amplified patterns depend on the details of the connectivity and are best revealed by a Schur decomposition of the connectivity matrix.

We examine the dynamics of linear networks with patchy connectivity structures. With long-range patchy inhibition, about 25% of the eigenvalues of the connectivity matrix have strictly positive real parts. Since real parts must be smaller than one for stability, the overall magnitude of the connections, and therefore also the amount of transient amplification, need to be constrained. The presence of eigenvalues with real parts close to one also predicts dynamical slowing with a small number of modes (typically < 5) completely dominating the dynamics and limiting the representational power. If inhibition is kept local, as it seems to be in nature, the connectivity matrix has the nice property that all its eigenvalues have negative real parts. The network can thus be arbitrarily rescaled and yet remain stable. Strong amplification can coexist with fast network dynamics that yields a rich repertoire of transient spatial states.

Extensive simulations of the noise-driven linear networks confirm the theoretical insights. Importantly, we also ran large-scale simulations of conductance-based spiking neurons with sparse connectivity spatially organized as mentioned above. Despite the strong deviation from linearity, the network activity matches the linear predictions remarkably well, in terms of both speed and amplification of the Schur modes. We conclude that the activity in spatially organised networks is highly structured and can be described on the level of two-dimensional activity patterns. Local inhibition, but not long-range inhibition, enables rapid state switching on the time scale of a few milliseconds.

## References

[B1] Brunel N Dynamics of sparsely connected excitatory and inhibitory spiking neurons J Comput. Neurosci 2000 8 183 208 10.1023/A:1008925309027 10809012

[B2] Renart A de la Rocha J Bartho P Hollender L Parga N Reyes A Harris KD The asynchronous state in cortical circuits Science 2010 327 587 590 20110507 10.1126/science.1179850 PMC2861483

[B3] Voges N Schüz A Aertsen A Rotter S A modeler's view on the spatial structure of intrinsic horizontal connectivity in the neocortex Progr. Neurobiol 2010 92 277 292 10.1016/j.pneurobio.2010.05.001 20685378

[B4] Murphy B Miller KD Balanced amplification: a new mechanism of selective amplification of neural activity patterns Neuron 2009 61 635 648 19249282 10.1016/j.neuron.2009.02.005 PMC2667957

[B5] Trefethen LN Embree M Spectra and pseudospectra – The behavior of nonnormal matrices and operators 2005 Princeton University Press

